# Wallenda-Nmo Axis Regulates Growth via Hippo Signaling

**DOI:** 10.3389/fcell.2021.658288

**Published:** 2021-04-16

**Authors:** Xianping Wang, Hui Liang, Wenyan Xu, Xianjue Ma

**Affiliations:** ^1^Key Laboratory of Growth Regulation and Translational Research of Zhejiang Province, School of Life Sciences, Westlake University, Hangzhou, China; ^2^Westlake Laboratory of Life Sciences and Biomedicine, Hangzhou, China; ^3^Institute of Biology, Westlake Institute for Advanced Study, Hangzhou, China; ^4^Key Laboratory of Structural Biology of Zhejiang Province, School of Life Sciences, Westlake University, Hangzhou, China

**Keywords:** Wnd, Hippo signaling, Nmo, growth, *Drosophila*

## Abstract

Both Hippo signaling pathways and cell polarity regulation are critical for cell proliferation and the maintenance of tissue homeostasis, despite the well-established connections between cell polarity disruption and Hippo inactivation, the molecular mechanism by which aberrant cell polarity induces Hippo-mediated overgrowth remains underexplored. Here we use *Drosophila* wing discs as a model and identify the Wnd-Nmo axis as an important molecular link that bridges loss-of-cell polarity-triggered Hippo inactivation and overgrowth. We show that Wallenda (Wnd), a MAPKKK (mitogen-activated protein kinase kinase kinase) family member, is a novel regulator of Hippo pathways in *Drosophila* and that overexpression of Wnd promotes growth via Nemo (Nmo)- mediated Hippo pathway inactivation. We further demonstrate that both Wnd and Nmo are required for loss-of-cell polarity-induced overgrowth and Hippo inactivation. In summary, our findings provide a novel insight on how cell polarity loss contributes to overgrowth and uncover the Wnd-Nmo axis as an essential additional branch that regulates Hippo pathways in *Drosophila*.

## Introduction

Proper control of cell proliferation is fundamental for correct organ development, disruption of which would cause tumorigenesis. *Drosophila* imaginal discs, the precursor of the adult organ during the larval stage, are a powerful model to study tissue growth, and numerous signaling pathways essential for cell proliferation have been uncovered using this system (Gokhale and Pfleger, 2019; Gou et al., 2020), including the Hippo pathway, a key regulator of cell proliferation and organ size (Pan et al., 2018; Zheng and Pan, 2019). The core components of the Hippo signaling pathway consist of serine/threonine kinases Hippo (Hpo) and Warts (Wts) and the transcriptional coactivator Yorkie (*Yki*) (Justice et al., 1995; Xu et al., 1995; Harvey et al., 2003; Pantalacci et al., 2003; Udan et al., 2003; Wu et al., 2003; Huang et al., 2005). Upon phosphorylation by Hpo, Wts is activated and subsequently phosphorylates *Yki* to restrict its nuclear entrance, thereby preventing the transcription of pro-proliferative target genes, including *expanded* (*ex*), *bantam* (*ban*), and *Cyclin E* (*cycE*) (Pan, 2010; Snigdha et al., 2019; Zheng and Pan, 2019). Recent advances on this evolutionary conserved pathway have revealed comprehensive roles of Hippo signaling in regulating a wide range of biological functions, ranging from cell adhesion and mechanical tension to regeneration and immune surveillance (Fallahi et al., 2016; Chen, 2019; Ma et al., 2019; Zheng and Pan, 2019).

Cell polarity maintenance is essential for tissue homeostasis, and dysregulation of apical-basal polarity has been linked to various developmental disorders and cancer (Wodarz and Nathke, 2007). Studies in *Drosophila* have revealed that several apical-basal cell polarity modules regulate tissue growth via the Hippo pathway, including lethal-2-giant larvae (Lgl)-atypical protein kinase C (aPKC), Crumbs (Crb), and Scribbled (Scrib)/Disc large (Dlg) (Richardson and Portela, 2017). We recently identified the E3 ubiquitin ligase POSH (Plenty of SH3s) as a key regulator of Hippo pathway, which integrates the signal from the cell polarity protein Crb to negatively regulate Ex (Expanded)-mediated Hippo activation in *Drosophila* (Ma et al., 2018). We also identified Wallenda (Wnd), a member of mitogen activated protein kinase kinase kinase (MAPKKK) family, as an essential downstream component that links loss-of-polarity-induced cell invasion phenotype to JNK pathway activation (Ma et al., 2016). Although recent studies on *MAP3K13*, the human homolog of Wnd, have revealed its tumor promoting roles in head and neck squamous cell carcinoma (HNSCC) and hepatocellular carcinoma (HCC) patients (Edwards et al., 2017; Zhang et al., 2020), the mechanism by which Wnd/MAP3K13 regulates tissue growth remains underexplored.

In this study, we uncovered Wnd as a novel regulator of Hippo signaling. Our results showed that overexpression of Wnd induces cell proliferation in the *Drosophila* wing imaginal disc via inactivating the Hippo signaling. Knockdown of *wnd* impedes *Rho1* expression or loss-of-polarity induced Hippo inactivation. We further identified Nemo (Nmo) as an essential downstream mediator of Wnd in regulating Hippo signaling. Our data demonstrate that Wnd-Nmo represents a new axis that bridges cell-polarity-loss-induced growth and Hippo pathway inactivation in *Drosophila*.

## Results and Discussion

### Wnd/MAP3K13 Negatively Regulates Hippo Signaling

**FIGURE 1:**
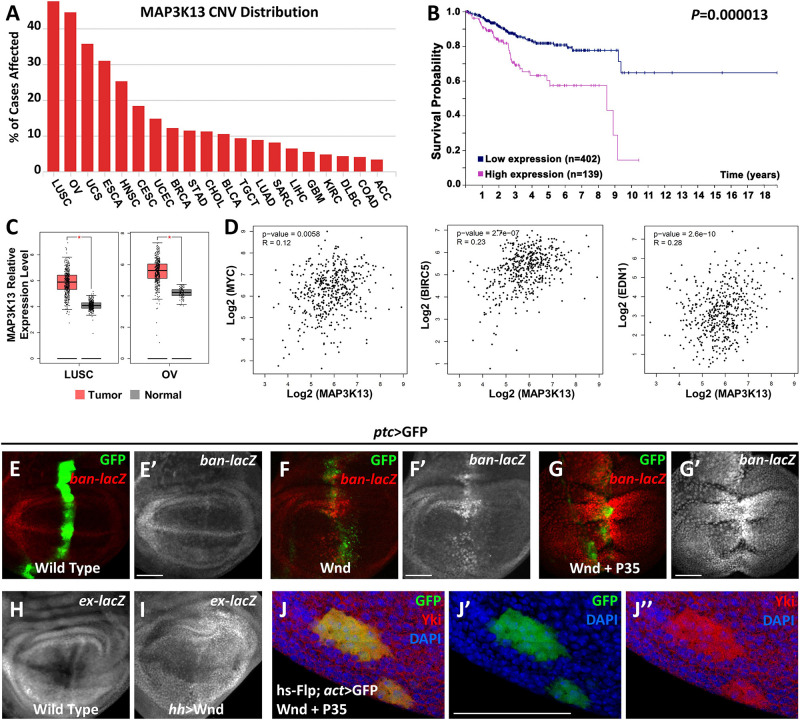
MAP3K13/Wnd activates expression of Hippo pathway target genes. **(A)** The distribution of copy number variation (CNV) of *MAP3K13* across different cancer types. **(B)** Analysis of the prognostic significance of high and low expression level of *MAP3K13* in endometrial cancer. **(C)** Box plots showing the relative expression level of *MAP3K13* in lung squamous cell carcinoma (LUSC) and ovarian serous cystadenocarcinoma (OV). **(D)** Expression correlation between *MAP3K13* and *MYC*, *BIRC5*, or *EDN1*, three target genes downstream of the transcriptional co-activators YAP/TAZ, in LUSC. **(E–J″)** Wing imaginal discs from third instar larva are shown. Compared with wild type controls **(E,E′)**, expression of Wnd for 2–3 days **(F,F′)** or co-expression of *wnd* and *P35* **(G,G′)** driven by *ptc*-Gal4 (GFP positive area) elevates *ban* expression level. Expression of Wnd in the posterior region of wing disc elevates *ex* expression level **(I)**, compared with wild type controls **(H)**. **(J–J″)** *Yki* nuclear localization is increased in heat shock-induced clones expressing *wnd*. Scale bars, 50 μm.

Our previous studies have revealed that both *Rho1* and Wnd are essential for tumorigenesis by inducing JNK signaling mediated cell death and cell invasion in wing imaginal discs (Ma et al., 2015b, 2016). Interestingly, we noticed that *Rho1*-induced actin accumulation could only be impeded by depletion of Wnd but remained unaffected by complete inhibition of JNK signaling, suggesting that Wnd has additional roles other than the JNK pathway activation. To further explore the role of Wnd in regulating tumorigenesis, we examined the potential role of MAP3K13 (human homolog of Wnd) through data mining using several open-access online servers. We found that copy number variants (CNV) of *MAP3K13* are dysregulated in various human cancers (Figure 1A), especially in lung squamous cell carcinoma (LUSC) and ovarian serous cystadenocarcinoma (OV). Accordingly, transcripts of *MAP3K13* are significantly up regulated in LUSC and OV (Figure 1C) and the high expression level of *MAP3K13* is correlated with low survival probability in endometrial cancer patients (Figure 1B). Together, these results indicate that *MAP3K13* is a potential oncogene.

Next, we tried to further address the function of Wnd in tissue growth control *in vivo* using *Drosophila*. Given that ectopic *wnd* expression can induce the up-regulation of *wingless* (*wg*) and *cycE* (Ma et al., 2016), two known downstream target genes of the Hippo pathway, we tested the possibility that *wnd* could regulate the Hippo pathway in *Drosophila*. We examined the transcriptional change of two additional Hippo signaling reporters, *ban* and *ex* (Cho et al., 2006; Thompson and Cohen, 2006), and found that upon Wnd overexpression under *ptc* promoter along the anterior-posterior boundary of wing imaginal discs, an obvious up-regulation of *ban-lacZ* was seen (Figures 1F,F′), compared with the wild-type control (Figures 1E,E′). Given that ectopic expression of Wnd also leads to apoptosis as reported previously (Ma et al., 2015b), we inhibited apoptosis simultaneously by co-expression of *P35* with *wnd* and observed a stronger up-regulation of *ban-lacZ* (Figures 1G,G′). Similarly, overexpression of *wnd* by *hh-Gal4* in the posterior region of wing imaginal discs significantly up-regulated *ex-lacZ* level (Figures 1H,I). More importantly, we also observed strong nuclear *Yki* localization in heat-shock-induced *wnd* expressing clones compared with endogenous control outside the GFP positive clones (Figures 1J–J″). Furthermore, our data mining results suggested that the up-regulation of *MAP3K13* in LUSC is positively correlated with increased expression of *MYC*, *BIRC5*, and *EDN1* (Figure 1D), three known target genes downstream of YAP/TAZ (Choi et al., 2018), indicating a potential conserved role of *MAP3K13* in regulating Hippo pathway in human cancers. Interestingly, we also observed an increased level of cytoplasmic *Yki*, suggesting that there is an overall increase of the cellular *Yki* amount, which we could not explain at this point, and for which further investigation is required to dissect the underlying mechanism. Therefore, we conclude that Wnd enhances *Yki* nuclear localization and up-regulates Hippo pathway target genes.

### Wnd Acts Downstream of Cell Polarity Loss-Rho1 Axis

**FIGURE 2:**
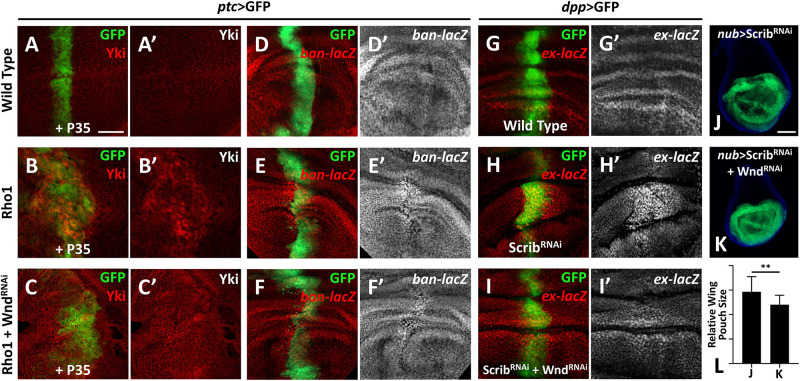
Wnd is essential for *Rho1* activation and cell polarity loss-induced Hippo inactivation. **(A–K)** Wing imaginal discs from third instar larva with genes or RNA is expressed in GFP positive area are shown. **(A–C′)** Compared with wild type controls **(A,A′)**, expression of *Rho1* together with *P35* enhances Yki nuclear localization **(B,B′)**, which is rescued by knocking down *wnd* **(C,C′)**. **(D–F′)** Compared with wild type controls (D,D′), expression of *Rho1* induced *ban* upregulation **(E,E′)** is suppressed by knocking down *wnd* **(F,F′)**. **(G–I′)** Compared with wild type controls **(G,G′)**, *scribble* (*scrib*) knockdown upregulates *ex* transcription **(H,H′)** and this is impeded by *wnd* knockdown **(I,I′)**. **(J)** Knocking down *scrib* in the wing pouch region causes tissue overgrowth **(J)**, which is suppressed by *wnd* knockdown **(K)**. Statistical analysis of **(J,K)** is shown in **(L)**. Scale bars for **(A–I′)**, 50 μm; for **(J,K)**, 100 μm. **P < 0.01. (A Student *t*-test was used to calculate statistical significance, mean + *SD*, *n* = 15).

Our previous study revealed a physical interaction between Wnd and the Rho GTPase *Rho1* in regulating cell invasion (Ma et al., 2016), and we identified *Rho1* as an important regulator of Hippo signaling-mediated growth (Ma et al., 2015a). These clues raised the possibility that Wnd and *Rho1* are somehow linked in the regulation of the Hippo pathway. Therefore, we investigated whether Wnd is also required for *Rho1*-mediated Hippo inactivation in *Drosophila*. Overexpression of *Rho1* with *P35* by *ptc-Gal4* in wing imaginal discs induces obvious cell proliferation and enhanced *Yki* nuclear localization (Figures 2B,B′), both of which are significantly impeded by knockdown of *wnd* (Figures 2C,C′). Consistently with this, we found that compared with the wild type control (Figures 2D,D′), knockdown of *wnd* also reduces *Rho1* overexpression-induced *ban-lacZ* up-regulation (Figures 2E–F′ and Supplementary Figure 2), suggesting that Wnd is required for Rho1-induced Hippo signaling inactivation.

Loss of *scrib* has been reported to upregulate *Yki* target genes (Verghese et al., 2012). To test whether Wnd is also required for *scrib*-loss induced Hippo inactivation, we monitored *ex-lacZ* level in wing imaginal discs. Knockdown of *scrib* by *dpp-*Gal4 induces evident upregulation of *ex-lacZ* (Figures 2H,H′), in comparison with the wild type control (Figures 2G,G′), while knockdown simultaneously of *wnd* reduces the *ex-lacZ* level to a certain degree (Figures 2I,I′). Additionally, knockdown of *scrib* by *nub*-Gal4 leads to significant overgrowth of the wing pouch region (Figure 2J), which is also inhibited by *wnd* knockdown (Figures 2K,L). In summary, these results indicate that Wnd acts downstream of cell polarity-loss-induced Hippo inactivation.

### *Nmo* Genetically Acts Downstream of *Wnd*

Next, we further dissected the potential mechanism by which *wnd* overexpression inactivates Hippo signaling. As a *MAPKKK*, logically, Wnd may activate certain protein kinase(s) that are known to genetically interact with Hippo signaling. However, the classical known kinases of the Hippo pathway are all negative regulators of *Yki*, suggesting that Wnd might act through a non-canonical kinase target to regulate the Hippo pathway. The only exception known so far is a MAPK family protein named Nmo, a serine/threonine protein kinase essential for cell death, planar cell polarity, neuronal function, and circadian clock regulation (Mirkovic et al., 2002; Merino et al., 2009; Yu et al., 2011; Collu et al., 2018). It is recently reported that Nemo-Like Kinase (*NLK*, human homolog of Nmo) phosphorylates YAP and subsequently positively regulate its transcriptional activity (Moon et al., 2017). It is worth noting that both Wnd and Nmo are essential for the overgrowth phenotype of neuromuscular junction (NMJ) caused by a mutation in a ubiquitin E3 ligase named Highwire (Collins et al., 2006; Wu et al., 2007; Merino et al., 2009), indicating a potential genetic interaction between *wnd* and *nmo*. Therefore, we examined whether Nmo is genetically involved in Wnd-mediated *Yki* activation.

**FIGURE 3:**
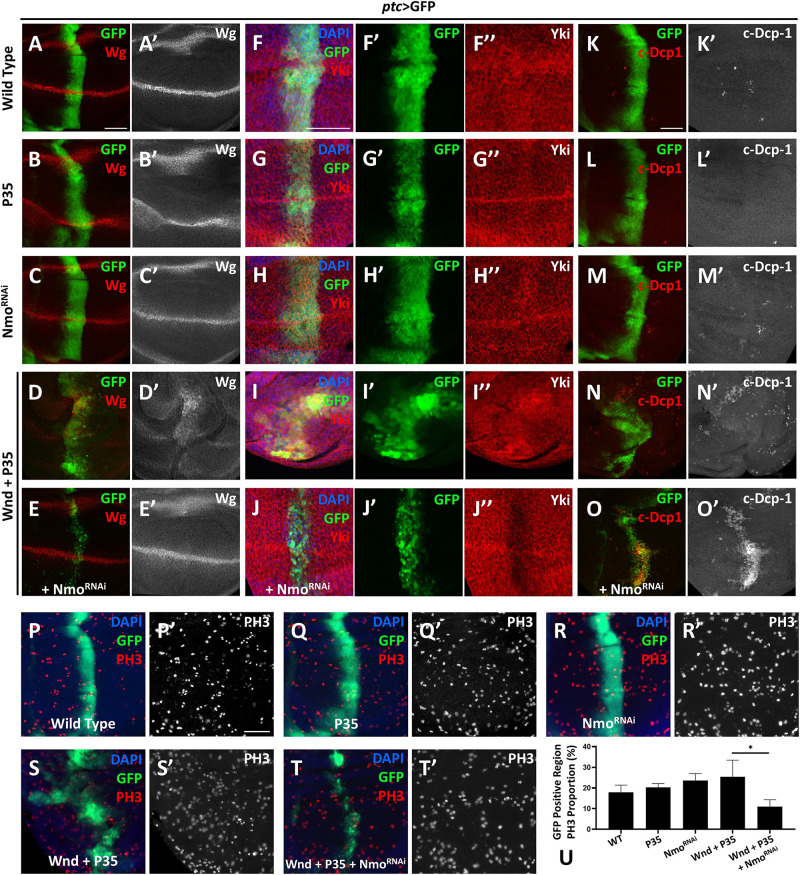
*Nmo* acts genetically downstream of *wnd*. Wing imaginal discs from third instar larva with genes or RNAi expressed in GFP positive area driven by *ptc*-Gal4 are shown. **(A–E′)** Compared with wild type **(A,A′)** or *P35* expression alone control **(B,B′)**, co-expression of *wnd* and *P35* elevates Wingless (Wg) level **(D,D′)**, which is rescued by knocking down Nmo **(E,E′)**, while *nmo* knockdown alone shows little effect **(C,C′)**. **(F–J″)** Compared with wild type **(F–F″)** or *P35* expression alone control **(G–G″)**, co-expression of *wnd* and *P35* enhances *Yki* nuclear localization, which is completely rescued by *nmo* knockdown **(J–J″)**, while Nmo knockdown alone has little effect **(H–H″)**. **(K–O)** Compared with wild-type controls **(K,K′)** or *P35* expression alone controls **(L,L′)**, co-expression of Wnd and *P35* induce non-autonomous apoptosis (shown by c-Dcp-1 staining) throughout the wing disc **(N,N′)**. Although *nmo* knockdown alone does not affect apoptosis rate **(M,M′)**, upon Nmo knockdown under Wnd and *P35* co-expression background, fewer apoptosis signals are found outside GFP positive region while severe apoptosis is detected in an autonomous manner **(O,O′)**. **(P–T′)** PH3 staining images are shown to demonstrate cell proliferation. Compared with wild type **(P,P′)** or *P35* expression alone control **(Q,Q′)**, co-expression of *wnd* and *P35* elevates cell proliferation rate **(S,S′)**, which is suppressed by knocking down *nmo* **(T,T′)**, while *nmo* knockdown alone does not show significant difference **(R,R′)**. Scale bars, 50 μm. **(U)** Statistic analysis of the relative PH3 + number in panel P–T. ^∗^*P* < 0.05 (unpaired *t-*test with Welch’s correction was used to calculate statistical significance mean + *SD*, *n* ≥ 4).

Given that *wnd* overexpression induces cell death by activating JNK pathway, we co-expressed *P35* to inhibit Wnd-induced cell death. Overexpression of *P35* alone by *ptc*-Gal4 leads to no change on Wg level comparing to the wild type (Figures 3A–B′), iwhile co-expression of *wnd* and *P35* and observed a significant up-regulation of *wg* expression along the *ptc* stripe (GFP positive) (Figures 3D,D′), which was dramatically reduced by simultaneously knocking down *nmo* (Figures 3E,E′). Strikingly, we found that *nmo* knockdown not only reduced the nuclear *Yki* accumulation induced by co-expression of *wnd* and *P35*, but also caused sharp decrease of *Yki* protein level (Figures 3I–J″), whereas knockdown of *nmo* alone did not cause obvious phenotypes (Figures 3C,C′,H–H″, and Supplementary Figure 4). Since the increased nuclear *Yki* localization causally leads to cell proliferation, we also examined cell proliferation by PH3 staining. We found that though there is no significant difference of proliferation rate in *P35* overexpression and *nmo* knockdown wing discs compared with the wild type control (Figures 3P–R′,U), the increased proliferation caused by *wnd* and *P35* co-expression was significantly suppressed by *nmo* knockdown (Figures 3S–U). Consistent with genetic interactions between *wnd* and *nmo* in *Drosophila*, we found that upregulation of *MAP3K13* in LUSC and OV are positively correlated with increased expression of NLK (Supplementary Figure 1). Yki level reduction would induce apoptosis by downregulating the expression of *diap1* (Huang et al., 2005). Compared with controls (Figures 3K,K″), overexpression of *P35* (Figures 3L,L″) or knockdown of *nmo* (Figures 3M,M″) has no significant changes on apoptosis. However, we also observed non-autonomous apoptosis when Wnd and *P35* are co-overexpressed (Figures 3N,N′), which is commonly seen when JNK signaling is hyperactivated (Uhlirova et al., 2005; Perez-Garijo et al., 2013; La Marca and Richardson, 2020). Surprisingly, we found that Nmo inhibition caused a shift toward a strong autonomous apoptosis increase in the *wnd* and *P35* co-expression wing discs (Figures 3O,O′). Given that loss of *Yki* activity facilitates cell death (Huang et al., 2005; Liu et al., 2016), we speculate that in the Wnd and *P35* co-expressing wing disc, *nmo* knockdown leads to the downregulation of *Yki* level and therefore increases apoptosis. Taken together, these data indicate that Nmo is an essential downstream effector of Wnd in regulating Hippo signaling.

### Nmo Is Essential for Impaired Cell Polarity or Rho1-Induced Hippo Inactivation

**FIGURE 4:**
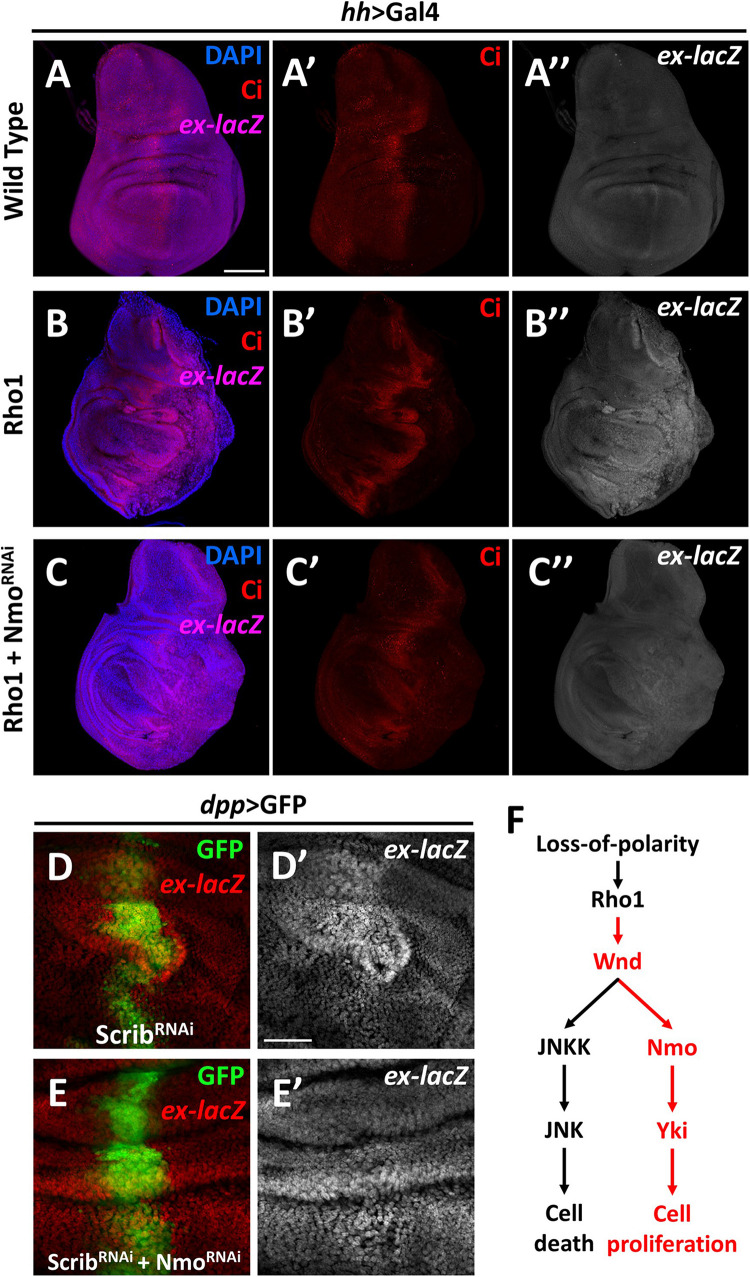
Nmo is essential for loss-of-cell polarity induced growth and *Yki* activation Wing imaginal discs from third instar larva are shown. **(A–C″)** Gene or *RNAi* is expressed driven by *hh*-Gal4 in the posterior region (opposite to ci-stained zone). Compared with wild type control **(A–A″)**, expression of *Rho1* elevates *ex-lacZ* expression level **(B–B″)**, which is suppressed by *nmo* knockdown **(C–C″)**. **(D–E′)** RNAi lines are driven by *dpp*-Gal4 in the GFP positive region. *scrib* knockdown caused *ex* transcription upregulation **(D,D′)** is rescued by *nmo* knockdown **(E,E′)**. **(F)** A schematic model showing the regulation of Hippo signaling by the Wnd-Nmo axis. Scale bars, 50 μm.

The data described above show that Nmo is required for Wnd-induced *Yki* activation, and Wnd is required for Rho1 activation and cell polarity-loss-induced Hippo inactivation. Next, we asked whether Nmo is also required for *scrib* loss and *Rho1* activation-induced tissue growth and Hippo inactivation. Expression of *Rho1* in the posterior region of wing discs under the control of *hh*-Gal4 autonomously up-regulates *ex* transcription and induces tissue overgrowth (Figures 4A–B″), which are significantly reduced by co-expression of *nmo* and RNAi (Figure 4C″ and Supplementary Figure 3). Consistently, we found that reducing Nmo activity significantly impeded *scrib*. *RNAi* induced *ex-lacZ* upregulation (Figures 4D′,E′). Together, these results indicate that Nmo is an essential regulator of impaired cell polarity and *Rho1*-induced Hippo inactivation in *Drosophila* wing disc.

In summary, our data here revealed a novel role of Wnd in promoting cell proliferation through inhibiting the Hippo pathway activity. We further demonstrated that Nmo, a MAPK family kinase, is required for the regulation of the Hippo pathway downstream of Wnd (Figure 4F). As a MAPKKK, Wnd is orthologous to both MAP3K13 and MAP3K12 in humans. Studies of these two proteins mainly focus on neuronal development, which is also the main topic of Wnd-related studies, while several studies on MAP3K13 also revealed its relation to tumor development (Han et al., 2016; Edwards et al., 2017; Chen et al., 2018; Wlaschin et al., 2018; Zhang et al., 2020). In this study, we genetically linked Wnd to the Hippo pathway for the first time and provided *in vivo* evidence that Wnd promotes cell proliferation in some contexts. Nmo (NLK) is involved in diverse developmental and cellular processes and was recently identified as a positive regulator of *Yki*/YAP activity (Moon et al., 2017; Daams and Massoumi, 2020). Despite its sequence characteristics as a MAPK, no MAPKK has been reported to phosphorylate NLK, and in contrast it can auto-phosphorylate itself. It is possible that at least in *Drosophila*, a Wnd-Nmo axis may exist. Interestingly, in accordance with our findings, a recent study identified both Wnd and Nmo in the same genetic screen, aiming to reveal the underlying mechanisms of Alk (Anaplastic lymphoma kinase) oncogenic signaling (Wolfstetter et al., 2020). It is also noteworthy that we found *wnd* and *P35* overexpression-induced growth phenotype can be significantly suppressed by co-expression of *wts* (Supplementary Figure 5), indicating that an additional regulating mechanism may exist downstream of Wnd. Therefore, we cannot exclude the possibility that Wnd-Nmo could act on *Yki* via the inactivation of Wts or other kinase(s), and further investigation is required to dissect the underlying mechanism of Wnd-mediated Hippo pathway regulation. Taking advantage of the *Drosophila* model, our findings here suggest the exciting prospect that similar mechanisms may exist in human cancer progression, and further investigation is required to elucidate the potential link between MAP3K13-*NLK* axis and polarity loss induced YAP/TAZ activation in tumorigenesis.

## Materials and Methods

### *Drosophila* Strains

*Drosophila* stocks were reared on standard media at 25°C unless otherwise indicated. For Figures 1F–I, *tub*-Gal80ts was used, flies were first raised at 18°C to restrict Gal4 activity for 5–6 days, then shifted to 29°C for 2–3 days to inactivate Gal80ts. The following strains were used for this study: *ptc*-Gal4, *UAS*-GFP, *UAS*-p35, *ban*-*lacZ*, *ex-lacZ*, *UAS*-Rho1 (#7334), and *UAS*-wnd*.RNAi* (#27525) were obtained from the Bloomington Stock Center. *UAS-scrib.RNAi* (v27424) and *UAS-nmo.RNAi* (v3002) were collected from the Vienna *Drosophila* Resource Center. *UAS*-Wts was a gift from Shian Wu (Nankai University, Tianjin, China), *UAS*-Wnd (Collins et al., 2006) was a gift from Aaron DiAntonio (Washington University, St. Louis, MO).

### Clonal Analysis

Flp-out ectopic expression clones in Figure 1J were generated by crossing *UAS*-Wnd; *UAS*-*p35* with y w *hs-*FLP; *act* > *y*^+^ > Gal4,*UAS*-GFP. Clones were induced at the second instar: heat shock for 10 min at 37°C 48–72 h after egg laying (AEL), dissection were performed 36 h after clone induction.

### Immunohistochemistry

Third instar larvae were dissected to collect their wing imaginal discs. Tissues were fixed with 4% formaldehyde and then washed with PBST (PBS + Triton, 1,000:3) for 5 min for 3 times at room temperature (RT). Block in PBST with Goat Serum (Solarbio SL038, 1:10) at RT for 30 min, then treat with 1st antibody at 4°C overnight. The following antibodies were used: rabbit anti-*Yki* (gist from Duojia Pan, 1:500); mouse anti-β-Gal (Promega, 1:500); mouse anti-wingless (DSHB 4D4, 1:100); rabbit anti-cleaved Dcp-1 (CST 9578, 1:100); Rat anti-Ci (DSHB 2A1 1:50); rabbit anti-Phospho-Histone H3 (Ser10) (CST 9701, 1:200). Next, wash the tissue with PBST for 10 min for 3 times at RT, then treat with the second antibody at RT for 2 h. Wash with PBST for 5 min for 3 times. For experiments involving nuclear localization of *Yki* and for Figure 4, additional DAPI staining was conducted by treating the tissue with DAPI (Beyotime C1,002) in PBST (1:1,000) at RT for 10 min. Finally, move the tissue onto a slide glass and mount with mounting media with DAPI (VECTASHIELD H-1,800) and then covered by cover glass.

### Imaging and Analysis

Wing discs were imaged with the Zeiss Axio Observer microscope. Image J (Fiji) was used to count PH3 the number of positive cells. All statistical analyses were performed using GraphPad Prism 8. The experiments are repeated for at least 4 times except for Figures 3F–G″, which is repeated for 3 times. Selected pictures are representative ones. Adobe Photoshop (22.1) was used to process and adjust images. Data were statistically analyzed by the Student *t*-test, the unpaired *t-*test with Welch’s correction, or the Kruskal-Wallis test, showed in bar graph as mean + *SD*.

### Database Analyses

CNV analysis in Figure 2A was processed on the Genomic Data Commons Data Portal^1^, a robust data-driven platform for access to cancer data for analysis, based on The Cancer Genome Atlas (TCGA) database. Only gain-of-number results are shown. Prognostic analysis in Figure 1B was conducted through The Human Protein Atlas^2^, a program aiming to map all the human proteins. This platform automatically found three cancer types that show significant difference in prognosis under low or high MAP3K13 expression level. The selected endometrial cancer result is a representative one. Expression analysis in Figure 1C and correlation analysis in Figure 1D was carried out by using the GEPIA^3^, an interacting web server to analyze RNA sequencing data provided by TCGA and Genotype-Tissue Expression (GTEx). We choose LUSC and OV for expression analysis and LUSC for correlation analysis because these two cancer types are the top two hits in the CNV analysis.

## Data Availability Statement

Publicly available datasets were analyzed in this study. This data can be found here: The Cancer Genome Atlas (TCGA) (https://tcga-data.nci.nih.gov/tcga/; dbGaP accession number: phs000178.v1.p1).

## Author Contributions

XM conceived the study. XW, HL, and WX performed the experiments. XW, HL, and XM analyzed the data and wrote the manuscript. All authors contributed to the article and approved the submitted version.

## Conflict of Interest

The authors declare that the research was conducted in the absence of any commercial or financial relationships that could be construed as a potential conflict of interest.

## Footnotes

1 https://portal.gdc.cancer.gov

2 https://www.proteinatlas.org

3 http://gepia.cancer-pku.cn/index.html
