# Supplementary material for: Wallenda-Nmo Axis Regulates Growth via Hippo Signaling
Source: Front Cell Dev Biol. 2021 Apr 16;9:658288. doi: 10.3389/fcell.2021.658288 (PMC8085559; doi:10.3389/fcell.2021.658288)
Supplement: Supplementary file 1 [file Data_Sheet_1.docx]

**Wallenda-Nmo Axis Regulates Growth via Hippo Signaling**

Xianping Wang, Hui Liang, Wenyan Xu, Xianjue Ma

**Supplemental Figures**


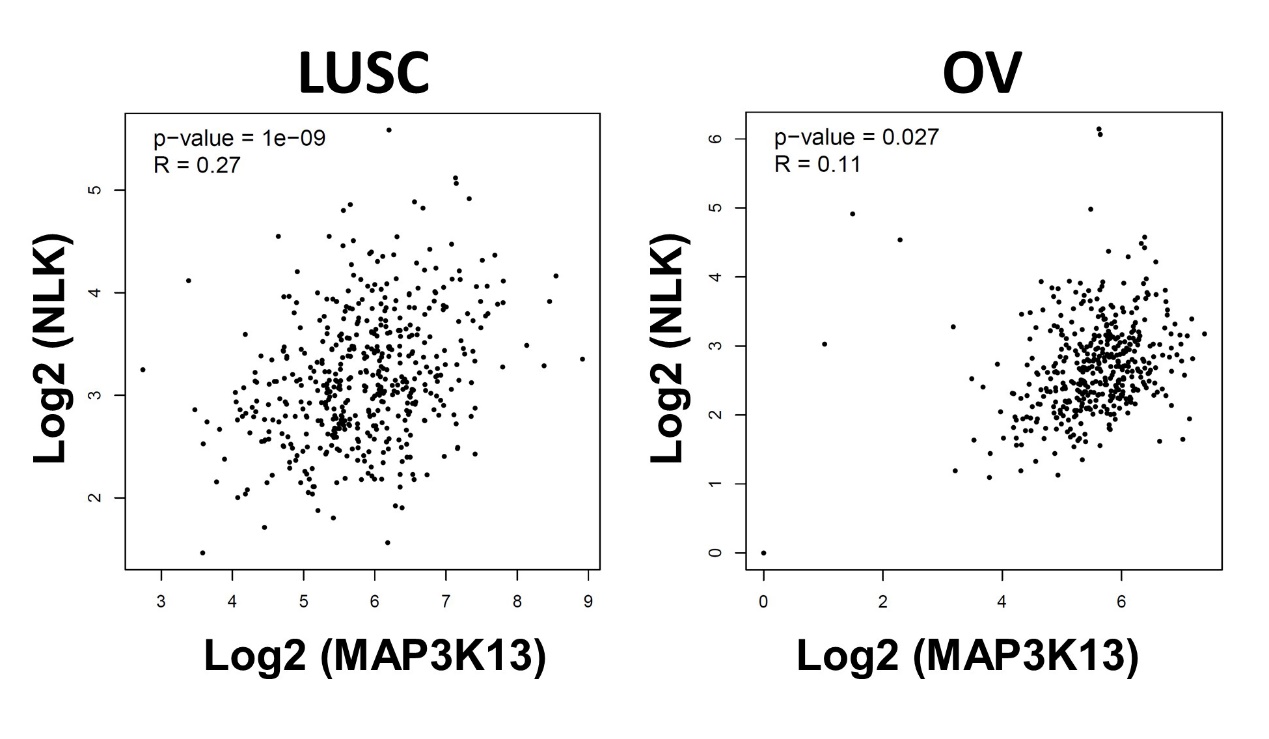


**Figure S1. The expression level of *MAP3K13* in LUSC and OV cancer is positively correlated with *NLK*.**

Positive correlation between *MAP3K13* and *NLK* (*nmo* homolog in human) in LUSC or OV cancer sample, based on TCGA database.


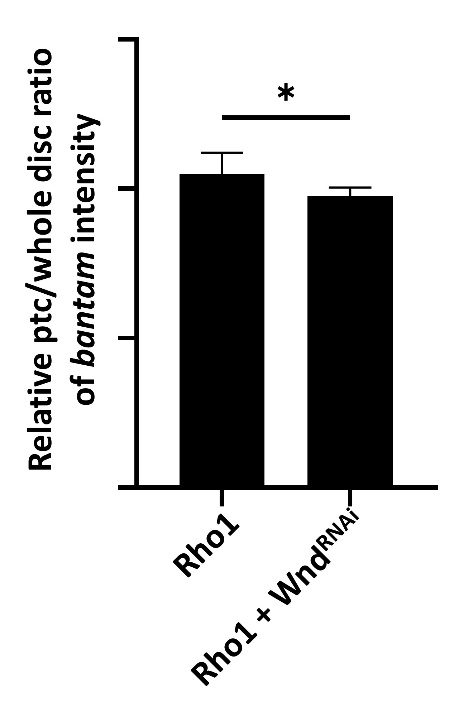
**Figure S2. Statistical analysis of Figure 2E-F’.**

Statistical analysis of Figure 2E-F’. Y axis shows the relative intensity of *ban-lacZ* in the ptc region compared with the whole disc. In the background of *Rho1* overexpression, the intensity of *bantam* reduces by knockdown of *Wnd*. *, *P*<0.05. (Student’s t-test was used to analyze the statistical significance, mean+s.d., n≥5).


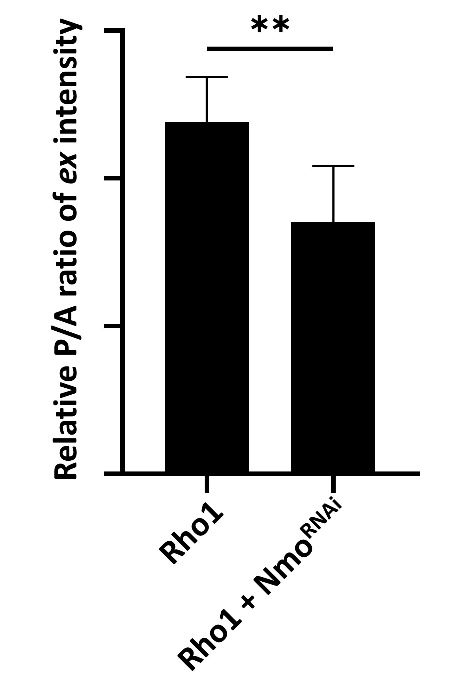
**Figure S3.** **Statistical analysis of Figure 4B-C”.**

Statistical analysis of *ex-lacZ* level in Figure 4B-C”. Y axis stands for the relative *ex-lacZ* intensity of posterior region (P) /anterior region (A) of wing discs. **, *P*<0.01. (Student’s t-test was used to analyze the statistical significance, mean+s.d., n≥5).

**Figure S4.** **Statistical analysis of Figure 3F-H”.**


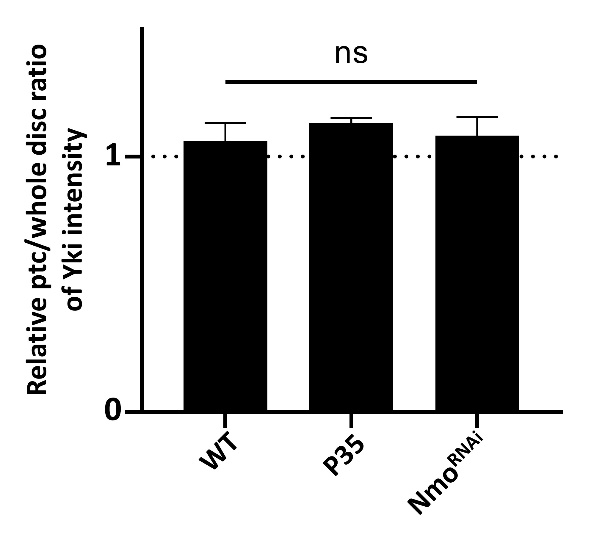
Statistical analysis of relative Yki level in Figure 3F-H’’. It shows a slight elevation in the Yki intensity in all three groups. Statistically, this elevation is not significant comparing all three groups. ns, not significant, p≥0.05. (Kruskal-Wallis test was used to analyze statistical significance, mean+s.d., n≥3)


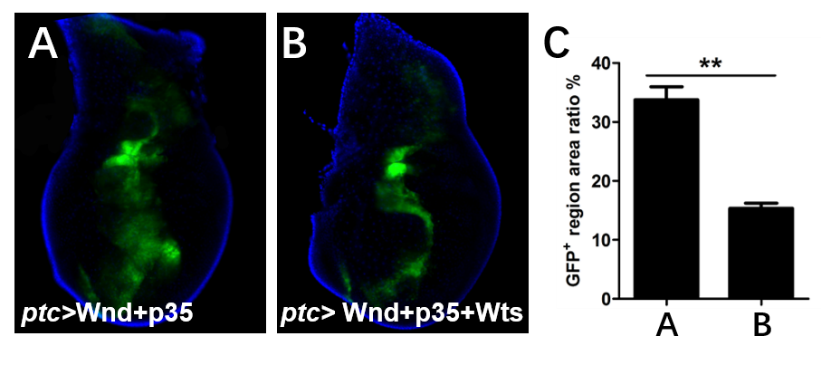


**Figure S5. Coexpression of *wts* suppresses *Wnd* and *P35* induced overgrowth.**

(A-B) Wing imaginal discs of third instar larva. *Wts* overexpression suppresses overgrowth of ptc region induced by co-expression of *Wnd* and *P35*. (C) Statistical analysis of A and B. Y axis stands for the relative size of GFP region in the wing discs. **, *P*<0.01. (Student’s t-test was used to analyze statistical significance, mean+s.d., n=3).
